# Supplementary material for: High mitochondrial diversity of domesticated goats persisted among Bronze and Iron Age pastoralists in the Inner Asian Mountain Corridor
Source: PLoS One. 2020 May 21;15(5):e0233333. doi: 10.1371/journal.pone.0233333 (PMC7241827; doi:10.1371/journal.pone.0233333)
Supplement: S1 Text — (DOCX) [file pone.0233333.s001.docx]

**S1 Text 1**

**High mitochondrial diversity of domesticated goats persisted among Bronze and Iron Age pastoralists in the Inner Asian Mountain Corridor**

Taylor R. Hermes^1,2^*, Michael D. Frachetti^3^, Dmitriy Voyakin^4,5^, Antonina S. Yerlomaeva^6^, Arman Z. Beisenov^6^, Paula N. Doumani Dupuy^7^, Dmitry V. Papin^8^, Giedre Motuzaite-Matuzeviciute^9^, Jamsranjav Bayarsaikhan^10^, Jean-Luc Houle^11^, Alexey A. Tishkin^12^, Almut Nebel^13^, Ben Krause-Kyora^13^, Cheryl A. Makarewicz^1,2^*

^1^Graduate School “Human Development in Landscapes”, Kiel University, Kiel, Germany

^2^Institute of Prehistoric and Protohistoric Archaeology, Kiel University, Kiel, Germany

^3^Department of Anthropology, Washington University in St. Louis, St. Louis, Missouri, United States of America

^4^Archaeological Expertise, LLC, Almaty, Kazakhstan

^5^International Institute for Central Asian Studies, Samarkand, Uzbekistan

^6^Margulan Institute of Archaeology, Almaty, Kazakhstan

^7^School of Humanities and Social Sciences, Nazarbayev University, Nur-Sultan, Kazakhstan

^8^The Laboratory of Interdisciplinary Studies in Archaeology of Western Siberia and Altai, Altai State University, Barnaul, Russia

^9^Department of Archaeology, Vilnius University, Vilnius, Lithuania

^10^National Museum of Mongolia, Ulaanbaatar, Mongolia

^11^Department of Folk Studies and Anthropology, Western Kentucky University, Bowling Green, Kentucky, United States of America

^12^Department of Archaeology, Ethnography and Museology, Altai State University, Barnaul, Russia

^13^Institute of Clinical Molecular Biology, Kiel University, University Hospital Schleswig-Holstein, Kiel, Germany

*Corresponding authors: [trhermes@gshdl.uni-kiel.de](mailto:trhermes@gshdl.uni-kiel.de) and [c.makaewicz@ufg.uni-kiel.de](mailto:c.makaewicz@ufg.uni-kiel.de)

**Descriptions of archaeological sites**

**Sampled Sites in the “Inner Asian Mountain Corridor”**

**Begash.** Begash is a multi-period settlement site located in the foothills of the Dzhungar Mountains (800 m a.s.l.) within a tributary river valley of the Koksu River in southeastern Kazakhstan. Earliest occupations so far identified at the site began by 2400 cal BC and span into the early 20^th^ century AD, which is supported by a robust radiocarbon chronology of 35 AMS radiocarbon dates clustered within six main occupational phases [1–3]. Recovered architecture includes low-lying, rectangular stone foundations, over which organic material (animal hides or felt) was likely pitched to form small tent-like dwellings [1]. The faunal assemblage from the site consists largely of domesticated sheep, goat, and cattle throughout the cultural sequence, while horses were only recovered at relatively low frequencies from layers dated to the early second millennium BC and subsequent periods [4]. Analysis of cementum annulations may indicate that the site was mostly occupied during winter with less frequent occupations in spring, summer, and fall [5]. Macrobotanical remains recovered from the site include domesticated wheat, barley, and broomcorn millet dated to the second half of the third millennium BC, but these early carbonized seed remains were concentrated in a human burial cist and do not inform on the economic use of these crops [2,6]. However, stable isotopic research on livestock skeletal remains indicates livestock were foddered with millet, revealing a substantial and localized investment in millet cultivation by 2300 cal BC as part of the initial transmission of this crop westward to the steppe zone from western China [3]. Bronze pins and other small implements were also recovered from second millennium layers of the site [1], and the ceramic assemblage of Begash suggests local production and also protracted cultural exchange networks spanning the Eurasian steppe zone and southern Central Asia throughout the site’s chronological sequence [7–9].

**Dali.** Dali is an Early and Middle Bronze Age site located on the Bayan-Zhurek plateau (1500 m.a.s.l. of the Dzhungar Mountains in eastern Kazakhstan, Dali is represented by multi-period settlement and burial complex. The first phase of the settlement, dated to the first quarter of the third millennium BC [3], is represented by a pit house and lean-to structure utilizing large in-situ boulders. This period of occupation is characterized by a highly fragmented faunal assemblage that yielded *MT-CYB* sequences of sheep and goat domesticated in the Near East, currently representing the earliest known pastoral economies using caprines in the eastern steppe region [3]. Importantly, some of these animals exhibit high carbon isotopic values in winter forming portions of tooth enamel, indicating seasonal millet foddering that took place several hundred years earlier than that observed at the first phase of Begash [3]. The second phase of Dali is dated to the first half of the second millennium BC [3], during which architecture radically changes to rectangular stone foundations similar to that recovered at Begash. In both phases of the Dali settlement, there is evidence of local bronze production, represented by copper ores, slags, and finished bronze implements, including jewelry and utilitarian pins. A detailed archaeological analysis of Dali, including ceramics, fauna, metallurgy, and burials, is forthcoming.

**Tasbas.** The multi-period Bronze Age settlement site of Tasbas is located at about 1 km away from Dali. The earliest phase of the site began at about 2800 cal BC, but is solely represented by a similar human cremation cist to that at Begash, in which domesticated wheat grains were recovered and directly dated to about 2500 cal BC [10]. The site appears to have been abandoned until the late second millennium BC [10], during which the site was reoccupied and crops of barley, wheat, millets, and legumes were locally cultivated and pastoralist livestock were herded [10,11]. The site remained in use until the Final Bronze Age (ca. 800 cal BC). Cementum annulations of sheep and goat teeth may indicate that the site was occupied year-round [5]. Ceramics recovered from the occupations dated to the second millennium BC were locally produced while exhibiting styles characteristic throughout Central Asia [7–9].

**Uch-Kurbu.** A Late Bronze Age cemetery site located in the Tian Shan mountains (1750 m.a.s.l.) of Kyrgyzstan, Uch-Kurbu is under active archaeological investigation. Preliminary results indicate late Bronze Age occupations by mobile pastoralists who were also engaged in crop cultivation [12]. The site largely consists of Bronze Age human burials with associated ritual pits that date to about 1700-1300 cal BC, and use of the site may have extended into the early Iron Age [12].

**Sampled Sites in the “Northern Eurasian Steppe”**

**Bozshakol.** The Late Bronze Age settlement of Bozshakol is located in northern Kazakhstan (Pavlodar Oblast) approximately 50km southwest from the Irtysh River. The site also functioned as an important metallurgical complex. Rapid expansion of modern mining activity necessitated a large-scale rescue excavation the site by Archaeological Expertise, LLC that exposed over 3,000 m^2^ of a cultural layer, of which a majority had already been destroyed. Archaeological excavations recovered abundant quantities of bronze slags, copper ore, bronze ingots, casting molds, and metallurgical kilns and ash lenses. Free-standing structures, associated with both domestic occupation and metallurgical activity, consist of clusters of rooms, each about 20 m^2^, and vestibule-like corridors. Sheep, goat, cattle, and horses were exploited, and wild species, such as wild boar and saiga, were occasionally hunted. Ceramics were also abundant in the cultural horizon and show diverse regional styles utilizing comb stamps and impressions of curved and horizontal lines that are characteristic of Late Bronze Age communities across northern Kazakhstan. A small burial complex is associated with the settlement complex but is likely dated to the Iron Age. New radiocarbon dates taken from goat bone collagen (n=2) indicate that the site occupation spans 1600-1400 cal BC, which broadly corresponds to the relative chronology based on material culture.

**Rublevo-6.** The late Bronze Age settlement of Rublevo-6 is located in the Kulunda Plain of southwestern Siberia (Russia) and is positioned between two present-day ribbons of relict pine forest. The site was occupied from the about 1400 to 1000 cal BC, based on radiocarbon dates taken on animal bone collagen and charcoals recovered from cultural layers [13]. Settlement architecture recovered from the site is represented by a rectangular dwelling with two rooms, likely constructed from timbers, which other recovered features include middens and metallurgical kilns for bronze production [14]. The ceramic assemblage of Rublevo-6 corresponds with technology and stylistic motifs of the Sargary-Alekseevka typology, using few and dispersed incisions and fingernail impressions primarily at the rims of vessels [14,15]. Sheep, goat, cattle and horses were exploited, and hunting of wild species such as moose, played a minor role in the subsistence economy [16].

**Air-Tau.** The settlement of Air-Tau is located in the Tavricheskое district of Eastern-Kazakhstan province at the confluence of the Kanayka and Dresvyanka rivers. Excavations were conducted from 1989 to 1991 and uncovered numerous stone foundations that were largely fragmentary and difficult to define clear structures, but these remains were organized along a narrow strip along an upper terrace of the Dresvyanka river. Two main categories of Middle Bronze Age ceramics were recovered in association with one another that date to the first half of the second millennium BC: Federov-Bishkul and Sargarin-Alekseev. Metallurgical artifacts include bronze knives and scepters, and copper ores and slags. Faunal skeletal remains were abundant in occupational horizons. While zooarchaeological analyses are ongoing, preliminary results indicate that subsistence strategies were based on pastoralist livestock.

**Zamiin-Utug.** The Zamiin-Utug cemetery is located in Züünkhangai, Uvs province, in northwestern Mongolia. The site dates to the Iron Age Xiongnu period (ca. 209 BCE to 93 CE) and contains 92 ring burials organized in three separate groupings. To date, a single burial has been excavated in each section.

**Intermediary sites relative to the NES and IAMC**

**Taldysai.** The settlement of Taldysai is located in the Karagandy province of central Kazakhstan near the confluence of the Ulken Zhezdy and Bala Zhezdy rivers, which is positioned about 200 km south of the NES and 700 km northwest of the IAMC. The site dates to the middle-late Bronze Age and functioned as a large-scale metallurgical production center, indicated by abundant smelting ovens with long air channels, in addition to ores, slags, and finished bronze objects [17]. The Middle Bronze Age occupation (ca. 1900-1700 cal BC) is associated with the Petrovka archaeological culture, which is in large part defined on the basis of ceramic decorations of highly regular incisions composing zigzags, wavy lines, and hatched triangles that were originally used in large settlements, such as Sintashta, in the Ural region [18]. The Late Bronze Age (ca. 1600-1400 cal BC) occupation of Taldysai is associated with the Sargary-Alekseevka archaeological culture, which reflects a new pattern of ceramic ornamentation (see subsection on Rublevo-6) [9]. Limited zooarchaeological data show exploitation of pastoralist livestock and occasional hunting of wild species, such as saiga and kulan [17].

**Myrzhik.** The settlement of Myrzhik is located in the Atasu microdistrict of western Karagandy province about 10km to the southwest of the Atasu settlement (see below) [19]. The site is dated to the second half of the second millennium BC and served as a production center for bronze metallurgy [20]. There are more than 30 depressions consistent with above-ground structures that have been buried since their abandonment, and excavations (c. 1970-80s) laid out over four depressions revealed stone foundations with occupational horizons yielding faunal skeletal remains, ceramic sherds consistent with Late Bronze Age communities of central Kazakhstan, and copper ores and slags [17]. Excavations conducted in 2016 and 2017 revealed a semi-subterranean structure containing a large metallurgical kiln with five smaller satellite kilns. Zooarchaeological analysis of the faunal remains recovered from the older excavations show exploitation of pastoralist livestock with a substantial emphasis on caprines (~75% number of identifiable specimens—NISP) [21].

**Atasu.** The settlement of Atasu is located in the Atasu microdistrict of western Karagandy province on the bank of the Atasu river, representing one of the largest settlements in central Kazakhstan. Excavations conducted since 1955 and continued until 1980 revealed over 400 structures used as dwellings and metallurgical workshops. The site contains two distinct occupational horizons. The earlier phase is dated to the second half of the second millennium BC based on Late Bronze Age ceramics that are characteristic of this period for central Kazakhstan [17]. The later phase is dated to the first century of the first millennium BC based on Final Bronze Age ceramics [17]. For both phases, numerous copper ores, ingots, and tools were recovered, in addition to abundant faunal skeletal remains. Limited zooarchaeological analysis of the faunal remains revealed subsistence on pastoralist livestock with a greater occurrence of horse bones than at Myrzhik (~20%, versus <5% NISP, respectively) [21].

**References**

1. Frachetti MD, Mar’yashev AN. Long-Term Occupation and Seasonal Settlement of Eastern Eurasian Pastoralists at Begash, Kazakhstan. J Field Archaeol. 2007;32: 221–242. doi:10.1179/009346907791071520

2. Frachetti MD, Spengler RN, Fritz GJ, Mar’yashev AN. Earliest direct evidence for broomcorn millet and wheat in the central Eurasian steppe region. Antiquity. 2010;84: 993–1010.

3. Hermes TR, Frachetti MD, Doumani Dupuy PN, Mar’yashev A, Nebel A, Makarewicz CA. Early integration of pastoralism and millet cultivation in Bronze Age Eurasia. Proc R Soc B. 2019;286. doi:10.1098/rspb.2019.1273

4. Frachetti MD, Benecke N. From sheep to (some) horses: 4500 years of herd structure at the pastoralist settlement of Begash (south-eastern Kazakhstan). Antiquity. 2009;83: 1023–1037. doi:10.1017/S0003598X00099324

5. Schmaus TM, Doumani Dupuy PN, Frachetti MD. Variability in seasonal mobility patterns in Bronze and Iron Age Kazakhstan through cementum analysis. Quat Int. 2019 [cited 21 May 2019]. doi:10.1016/j.quaint.2019.04.018

6. Spengler R, Frachetti M, Doumani P, Rouse L, Cerasetti B, Bullion E, et al. Early agriculture and crop transmission among Bronze Age mobile pastoralists of Central Eurasia. Proc R Soc B Biol Sci. 2014;281: 20133382. doi:10.1098/rspb.2013.3382

7. Frachetti MD. Multiregional Emergence of Mobile Pastoralism and Nonuniform Institutional Complexity across Eurasia. Curr Anthropol. 2012;53: 2–38. doi:10.1086/663692

8. Doumani PN, Frachetti M. Bronze Age textile evidence in ceramic impressions: weaving and pottery technology among mobile pastoralists of central Eurasia. Antiquity. 2012;86: 368–382. doi:10.1017/S0003598X00062827

9. Doumani P. Ceramic Technology of Bronze Age Nomadic Pastoralists of Semirech’ye, Kazakhstan. Doctoral Dissertation, Washington University in St. Louis. 2014.

10. Doumani PN, Frachetti MD, Beardmore R, Schmaus TM, Spengler RN, Mar’yashev AN. Burial ritual, agriculture, and craft production among Bronze Age pastoralists at Tasbas (Kazakhstan). Archaeol Res Asia. 2015;1–2: 17–32. doi:10.1016/j.ara.2015.01.001

11. Spengler RN, Frachetti MD, Doumani PN. Late Bronze Age agriculture at Tasbas in the Dzhungar Mountains of eastern Kazakhstan. Quat Int. 2014;348: 147–157. doi:10.1016/j.quaint.2014.03.039

12. Motuzaite Matuzeviciute G, Abdykanova A, Kume S, Nishiaki Y, Tabaldiev K. The effect of geographical margins on cereal grain size variation: Case study for highlands of Kyrgyzstan. J Archaeol Sci Rep. 2018;20: 400–410. doi:10.1016/j.jasrep.2018.04.037

13. Papin DV. Khronologiya pamyatnikov epokhi pozdnej bronzy stepnogo i lesostepnogo Altaya. Vestn Kemer Gos Univ. 2015;6: 135–138.

14. Papin DV, Fedoruk AC. Khozyaistvenno-kul’turnyi tsentr epokhi pozdnej bronzy Rublevo-VI. Arkheologicheskiye issledovaniya stepnoj Evrazii. Karaganda: Tengri Ltd.; 2013. pp. 129–149.

15. Papin DV, Loman VG, Stepanova NF, Fedoruk AC. Rezultaty tekhniko-tekhnologicheskogo analiza keramicheskogo kompleksa poseleniya epokhi pozdnej bronzy Rublevo-VI. Teor Prakt Arkheologiskikh Issled. 2015;2: 115–143.

16. Kiryushin YuF, Kosintsev PA, Papin DV, Fedoruk AC. Voprosy khozyajstvennoj deyatel’nosti naseleniya stepnogo Ob’-Irtysh’ya v epokhu pozdnej bronzy. In: Kiryushin YuF, editor. Khozyajstvenno-kul’turnye traditsii Altaya v epokhu bronzy. Barnaul: Izd-vo Alt. un-ta; 2010. pp. 112–127.

17. Artyukhova OA, Kurmankulov Zh, Yermolayeva A, Erzhanova A. Kompleks pamyatnikov v urochische Taldysai. Almaty: Margulan Institute of Archaeology; 2013.

18. Koryakova L, Epimakhov AV. The Urals and Western Siberia in the Bronze and Iron Ages. Cambridge: Cambridge University Press; 2007.

19. Beisenov AZ. Pamyarniki verkhov’ev reki Atasu v tsentral’nom Kazakhstane. Vestn Tomsk Gos Univ. 2015;34: 111–122. doi:10.17223/19988613/35/17

20. Kadyrbaev MK, Kurmankulov Zh. Kul’tura drevnikh skotovodov i metallurgov Sary-Arki. Alma-Ata: Gylym; 1992.

21. Outram AK, Kasparov A, Stear NA, Varfolomeev V, Usmanova E, Evershed RP. Patterns of pastoralism in later Bronze Age Kazakhstan: new evidence from faunal and lipid residue analyses. J Archaeol Sci. 2012;39: 2424–2435. doi:10.1016/j.jas.2012.02.009
