# Supplementary figures and images for: High mitochondrial diversity of domesticated goats persisted among Bronze and Iron Age pastoralists in the Inner Asian Mountain Corridor
Source: PLoS One. 2020 May 21;15(5):e0233333. doi: 10.1371/journal.pone.0233333 (PMC7241827; doi:10.1371/journal.pone.0233333)

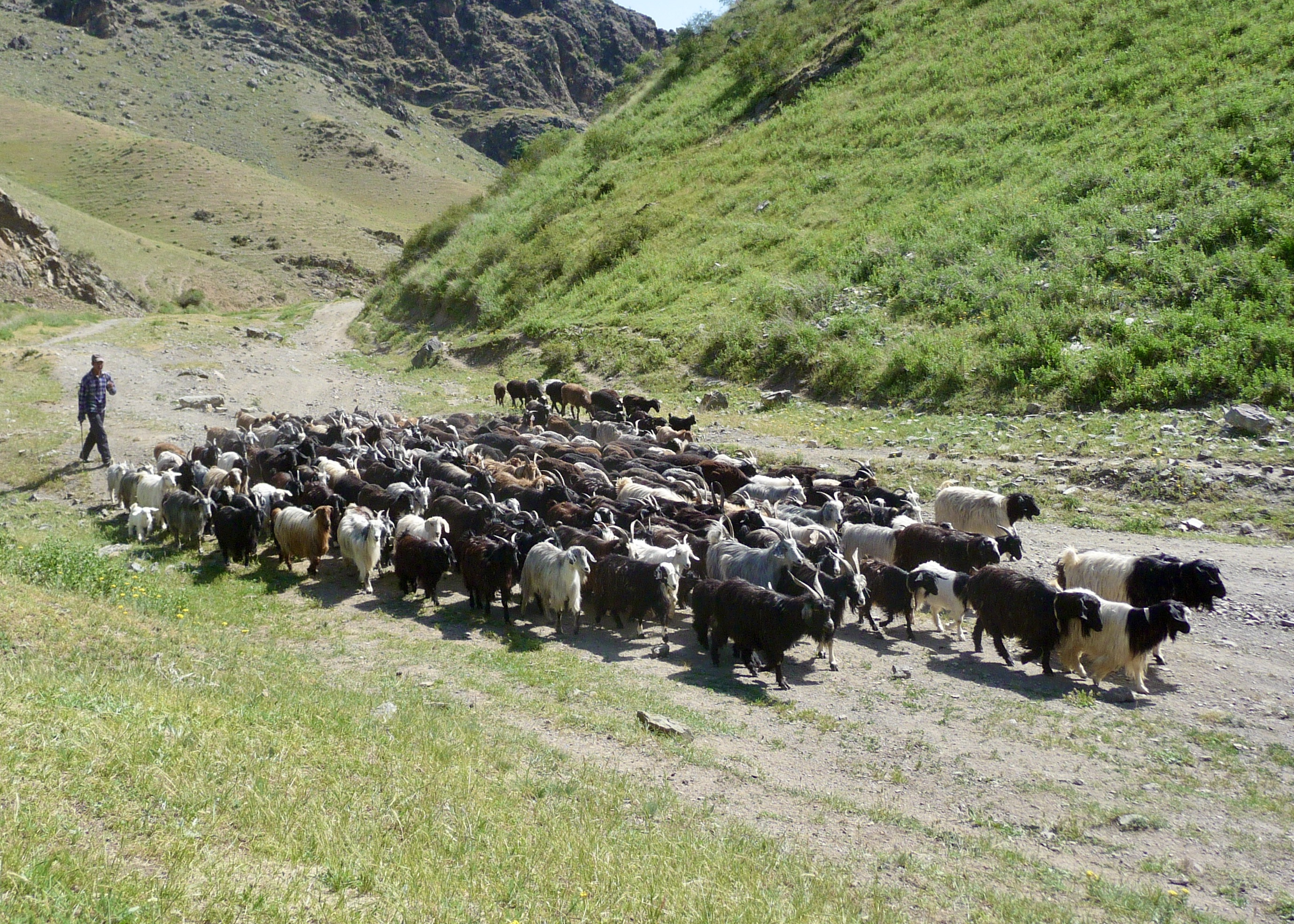

Supplement: S1 Fig — (TIF) [file pone.0233333.s007.tif]
